# Effects of simultaneous nicotine and alcohol use in periodontitis progression in rats: A histomorphometric study

**DOI:** 10.4317/jced.51047

**Published:** 2013-04-01

**Authors:** Daniel F. Pereira Vasconcelos, Marco A. Dias da Silva, Marcelo Rocha Marques, Cristina Gibilini, Any C. Cardoso Guimarães Vasconcelos, Silvana Pereira Barros

**Affiliations:** 1 Division of Histology and Embryology, School of Biomedicine, Federal University of Piauí, Parnaíba-PI, Brazi; 2 Division of Histology and Embryology, Federal University of Campina Grande, Patos-PB, Brazil; 3 Department of Morphology, Division of Histology, School of Dentistry at Piracicaba, University of Campinas, Piracicaba-SP, Brazil; 4 Department of Public Health Dentistry, School of Dentistry at Piracicaba, University of Campinas, Piracicaba-SP, Brazil; 5 Center for Oral and Systemic Diseases, Department of Periodontology, UNC School of Dentistry, University of North Carolina at Chapel Hill, USA

## Abstract

Objective: The aim of this study was to evaluate the effects of alcohol and nicotine, when used alone or simultaneously, on the alveolar bone loss area resulting from ligature-induced periodontitis in rats. Study design: Forty adult male rats received a cotton ligature in the first lower molar sulcular area, and the animals were randomly assigned to different treatments (n = 10, each group) including daily peritoneal injections of saline solution (group A), submitted to self-administration of alcohol 25% (group B), nicotine solution in concentration 0.19 μl/ml (group C), and nicotine solution in concentration 0.19 μl/ml plus self-administration of alcohol 25% (group D). Five weeks later, the animals were sacrificed, and the samples were routinely processed for semi-serial decalcified sections. Results: Ligated teeth showed more alveolar bone loss than unligated ones (p < 0.05). Unligated teeth showed no significant differences between each other (p > 0.05). Analyses between the ligated teeth showed that the group C (nicotine) or group B (alcohol 25%) each had increasing alveolar bone loss in the furcation area, and the simultaneous combination alcohol and nicotine (group D) intensified these effects (p < 0.05). Conclusion: The results suggest that the simultaneous combination of alcohol and nicotine have a synergistic effect in the progression of periodontitis, evidenced by increased furcation region bone destruction in periodontal disease in rats.

** Key words:**Alveolar bone loss, periodontitis, nicotine, alcohol, rats.

## Introduction

Destructive periodontal disease affects numerous people around the world (1). However, individuals respond differently to progressive periodontitis. One explanation for this difference may include genetic factors (2). It is widely accepted that the primary cause of periodontitis is bacterial infection (3). However, other factors may interfere with the development of destructive periodontal disease.

A recent investigation demonstrated that alcohol enhances periodontal inflammatory markers and exacerbates the development and progression of periodontitis (4). Another risk factor for periodontitis is cigarette smoking. Research has shown that nicotine is a toxic substance in tobacco smoke (5).

The effects of both nicotine (6) and alcohol (7) separately are harmful to periodontal tissues. These negative effects of alcohol on bone have been explained thought alcohol directly affects the number and activity of the osteoblasts and osteoclasts as well as it increases osteocyte apoptosis, further changes to cell differentiation may be responsible for the low bone mass and are associated with increased fat accumulation in the bone marrow (8).

Besides the effects of alcohol, studies have demonstrated an inhibition of proliferation, extracelular matrix production, and attachament of human gingival fibroblast, in addition to increased colagenase activity in the presence of nicotine (9), but little is known about the combined effects of alcohol and nicotine on periodontal tissues when used simultaneously.

The objective of this study was to evaluate the influence of simultaneous nicotine and alcohol use on furcation area bone loss rate in induced periodontitis in rats.

## Material and Methods

- Animals 

Forty adult male Wistar rats, weighing an average of 280 g, were used in this study. The animals were separated randomly into four groups (10 rats per group) and kept in cages in an animal room having a photoperiod of 12 h of light with access to industrial food, close to 110 Kcal/kg of body weight (10), and water was provided “ad libitum.” Room temperature was kept at between 22-25°C, and animals were allowed one week to adapt to the environment. The University of Campinas Institutional Animal Care and Use Committee approved the protocol under number 193-1/ UNICAMP-CEEA-IB-UNICAMP.

- Experimental Design

**Figure 1:**
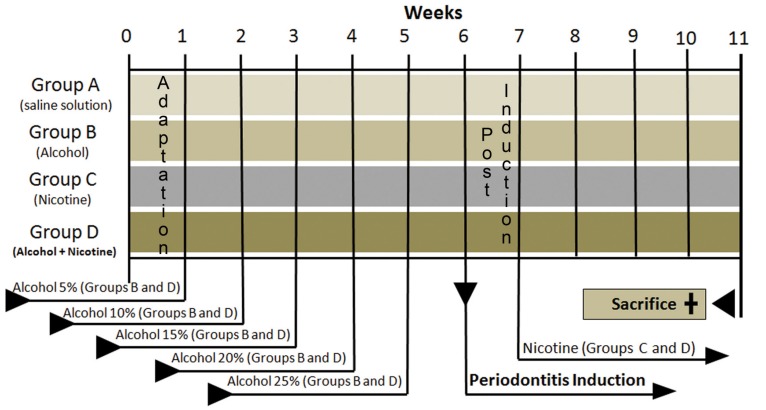
Experimental design illustrating the adaptation time, beginning treatments (alcohol and nicotine), periodontitis induction, and the time of sacrifice.

Twenty rats were submitted to self-administration of 25% alcohol. Alcohol doses were gradually introduced into the water by weekly, successive 5% increases in the concentration until a final concentration of 25% was obtained. The others twenty rats were given only filtered water. After five weeks, general anesthesia was induced by intramuscular administration with a solution of 13 mg/Kg of 2% xylazine hydrochloride (Rompum-Bayer-São Paulo, SP, Brazil) and 33 mg/Kg of ketamine (Francotar-Virbac-Roseira, SP, Brazil). To induce periodontitis, one of the mandibular first molars of each rat was randomly assigned to receive a cotton ligature around the cervical region (11). The ligature was knotted on the mesial side of the tooth. The contralateral tooth was left unligated to serve as control. Seven days later, the animals were randomly assigned to one of four treatment groups (Fig. 1), including the following daily intraperitoneal injection schemes.

group A, 2 μl/g body of weight of saline solution; group B, 2 µl/g body of weight of saline solution and self-administration of 25% alcohol; group C, 2 µl/g body weight of nicotine solution with 0.19 µl/ml of saline solution; and group D, 2 µl/g body weight of nicotine solution with 0.19 µl/ml of saline solution and self-administration of 25% alcohol. Thirty days after the beginning of nicotine administration (Fig. 1), the animals were sacrificed by cervical dislocation and jaws were processed (6). The experimental design is illustrated in figure 1.

- Histometrical evaluation

**Figure 2:**
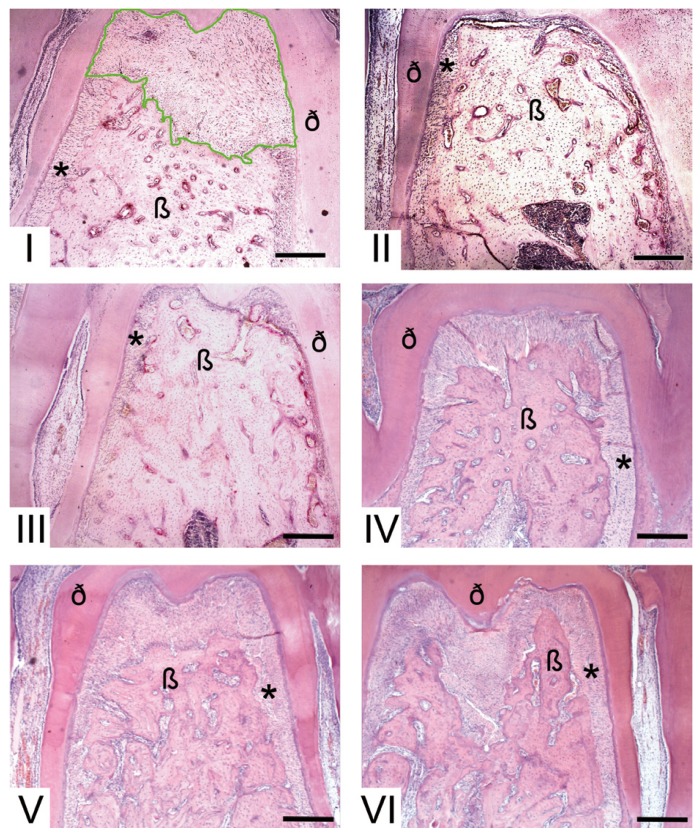
Photomicrography illustrating the furcation region of the molar teeth from different groups. I. Ligated tooth (periodontitis) with demarcation in green representing the measured area. II. Unligated tooth. III. Group A (ligated tooth). IV. Group B (ligated tooth). V. Group C (ligated tooth). VI. Group D (ligated tooth, * = periodontal ligament area, ß = bone, ð = dentine, bar = 300 µm at an original magnification 40x; hematoxylin and eosin).

The area of bone loss was assessed and the periodontal ligament between the bone crest and cementum surface was obtained using an ocular lens (10x) coupled to a 4x objective as demonstrated in figure 2. Paraffin serial sections were measured using an image analysis system (Image-Pro, Media Cybernetics, Silver Spring, MD). Ligated and unligated teeth were histometrically examined in 25 sections with 12 microns space between sections per hemi-jaw. Measurements from sections were averaged to allow intra- and intergroup analysis.

-Statistical Analysis

Tukey test was used to create multiple comparisons. The periodontal ligament area in the furcation region of the unligated teeth was compared among the groups. The same procedure was used in the ligated group. In addition, a paired t test was used for intragroup comparisons of the interradicular bone loss between ligated and unligated teeth. P values less than 0.05 were considered significant (BioEstat ver.5.0, Belém, PA, Brazil).

## Results

Clinically, signs of inflammation were observed in all ligated teeth. Statistical analysis (p > 0.05) of histometric values obtained from the bone loss areas revealed that there were no differences among the periodontal areas of unligated teeth (Fig. 2) from animals that were not exposed to drugs and also from the ones that received nicotine and/or alcohol. However, when periodontal tissue in the control unligated and contralateral ligated teeth (Fig. 2) groups were compared there was a significant difference (p < 0.05) in bone loss. These results demonstrate the disease was ligature-induced and that drug effects alone were not able to promote bone loss in the absence of inflammatory disease (Fig. 2).

**Figure 3:**
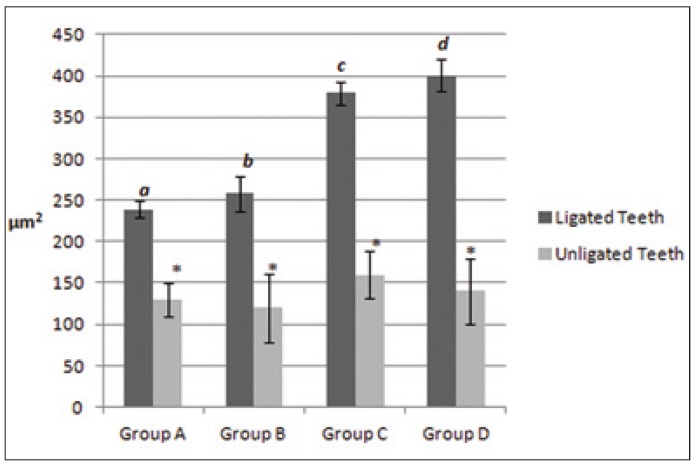
Shows the mean and standard deviation of the periodontal ligament and bone loss areas (µm2) around the ligated and unligated teeth. Different letters indicated a statistically significant difference (p < 0.05) and the asterisk denotes no statistical significance (p > 0.05).

Histometrically, intergroup analysis revealed greater bone loss (P < 0.05) in the ligated teeth in the animals that received either nicotine, alcohol, or simultaneous nicotine and alcohol administration [Fig. 2,3, group B = 250 ± 20 µm2, (Fig. 2); group C = 380 ± 10 μm2, (Fig. 2); group D = 400 ± 20 µm2, (Fig. 2)] compared to the ligated teeth in the animals which received saline solution (group A = 240 ± 10 µm2, Fig. 2). Moreover, the synergistic effect of simultaneous nicotine and alcohol administration on bone loss, group D, was statistically perceived (p < 0.05, Figs. 2,3).

## Discussion

Rats were chosen as the experimental model for this study because voluntarily consume alcohol similar to humans as well as being appropriate for the administration of drugs such as nicotine (12). However, the effect of simultaneous nicotine and alcohol use on periodontal tissues has not been well investigated.

In the present work, the heath of the animals and their liquid consumption was closely and systematically monitored throughout the duration of the experiment. Alcohol consumption can induced nutritional deficiency in rats (13), and this deficit could potentially lead to bone loss (14). However, rats treated with alcohol that were not ligated did not exhibit bone loss in the furcation area. Thus, the bone loss observed in these experiments likely came from the harmful effects of nicotine and alcohol in the presence of periodontal disease and are not due to malnutrition. Specifically, the present study demonstrates that simultaneous use of nicotine and alcohol increased bone loss in the furcation area of teeth in rats with induced periodontitis, whereas no effect was observed in unligated teeth.

Our study agrees with many previous reports that indicate that alcohol consumption has negative effects on periodontal tissues (4,7,8,15,16). Souza et al. (15) investigated the influence of alcohol on periodontitis induced by ligature and found that female rats that received 20% alcohol showed significantly greater bone loss (p < 0.05) in the furcation region when compared to those that received 10% alcohol (15). Our data corroborate those of Souza et al. (15). Data from group B (25% alcohol) demonstrates that bone loss (p < 0.05) in the furcation area was greater than group A (saline solution).

These negative effects of alcohol on bone have been explained by various plausible biological mechanisms. First, Maurel et al. (8) thought alcohol directly effects the number and activity of the osteoblasts and osteoclasts as well as it increases osteocyte apoptosis. Secondly, the changes observed may be modulated in part by a network of protein (Wnt) signaling pathways due to increased oxidative stress. Thirdly, changes to cell differentiation may be responsible for the low bone mass and are associated with increased fat accumulation in the bone marrow. Lastly, there may be direct effects of alcohol consumption on bone resulting from decreased calorie intake and a change of body composition.

Besides the effects of alcohol, another promoter of bone metabolism imbalance was investigated. Nicotine, is linked to the increased bone loss in periodontitis induced by ligature (6,17), and these results are corroborated by our data. Studies have demonstrated an inhibition of proliferation, extracelular matrix production, and attachament of human gingival fibroblast, in addition to increased colagenase activity in the presence of nicotine (9).

The effects of the consumption of alcohol or of nicotine use on bone tissues, separately, have been the subject of many investigations (4-10,12,14-16). However, a study with an experimental design similar to our study, found that the consumption of alcohol or nicotine could hinder or even impede the fixation and maintenance of bone implants in tibiae and femurs. The animals that received simultaneous nicotine and alcohol demonstrated the smallest volume of newly formed bone around the implants and the smallest mechanical bone resistance (18). It was observed that the effect of simultaneous nicotine and alcohol use was harmful to bone, which our data also demonstrates in the furcation area instead of the femur and tibia.

Based on the present results, we conclude that the treatment with alcohol or nicotine separately leads to negative effects on periodontal tissues. The simultaneous consumption of alcohol and nicotine intensified these effects, as well as increasing alveolar bone loss in furcation area, which were the most relevant data in this study. The present study might be useful to dental clinicians. The simultaneous consumption of alcohol and nicotine should be considered as an important factor for treatment and rehabilitation of patients.

The present study suggests that the simultaneous combination of alcohol and nicotine have synergistic negative effects on the progression of periodontitis, as measured by increased bone destruction in the furcation region in rats with periodontal disease.
